# Amine-Functionalized Sugarcane Bagasse: A Renewable Catalyst for Efficient Continuous Flow Knoevenagel Condensation Reaction at Room Temperature

**DOI:** 10.3390/molecules23010043

**Published:** 2017-12-24

**Authors:** Yanhui Qiao, Junjiang Teng, Shuangfei Wang, Hao Ma

**Affiliations:** 1 School of Chemistry and Chemical Engineering, Guangxi University, Nanning 530004, China; qyhmmc@gdupt.edu.cn; 2 College of Chemical Engineering, Guangdong University of Petrochemical Technology, Maoming 525000, China; tjjteng@gdupt.edu.cn; 3 College of Light Industry and Food Engineering, Guangxi University, Nanning 530004, China

**Keywords:** sugarcane bagasse, amine catalyst, Knoevenagel condensation, flow reaction, room temperature

## Abstract

A biomass-based catalyst with amine groups (–NH_2_), viz., amine-functionalized sugarcane bagasse (SCB-NH_2_), was prepared through the amination of sugarcane bagasse (SCB) in a two-step process. The physicochemical properties of the catalyst were characterized through FT-IR, elemental analysis, XRD, TG, and SEM-EDX techniques, which confirmed the –NH_2_ group was grafted onto SCB successfully. The catalytic performance of SCB-NH_2_ in Knoevenagel condensation reaction was tested in the batch and continuous flow reactions. Significantly, it was found that the catalytic performance of SCB-NH_2_ is better in flow system than that in batch system. Moreover, the SCB-NH_2_ presented an excellent catalytic activity and stability at the high flow rate. When the flow rate is at the 1.5 mL/min, no obvious deactivation was observed and the product yield and selectivity are more than 97% and 99% after 80 h of continuous reaction time, respectively. After the recovery of solvent from the resulting solution, a white solid was obtained as a target product. As a result, the SCB-NH_2_ is a promising catalyst for the synthesis of fine chemicals by Knoevenagel condensation reaction in large scale, and the modification of the renewable SCB with –NH_2_ group is a potential avenue for the preparation of amine-functionalized catalytic materials in industry.

## 1. Introduction

The Knoevenagel condensation, as one of the most classic methods for carbon-carbon bond formation in organic synthesis [1], is widely employed in the synthesis of high value-added chemicals such as therapeutic drugs, natural products, fine chemicals, carbocyclic and heterocyclic compounds of biological significance [2]. Normally, this reaction is proceeded in the presence of a basic catalyst through the nucleophilic addition of a carbanion to an electrophilic carbonyl group followed by a spontaneous dehydration process [3]; and the relatively weak base catalysts, viz., amine catalysts (such as aliphatic amines, urea and piperidine) or ammonia and their salts, are the preferred choices [4]. However, it is difficult to meet the increasing recoverability and recyclability criteria of these catalysts in a green and sustainable way in spite of the high catalytic activity. Thus, various solid catalysts with amine groups (–NH_2_) have been developed in recent decades.

In heterogeneous catalysis, the continuous reaction through the flow process has attracted much more attention [5]. Compared with traditional batch systems, the product yield, selectivity, quality and reproducibility are increased with continuous flow. Furthermore, the production costs, solvent consumption, and waste generation are also decreased with the continuous flow process [6,7]. The continuous flow processing can be regarded as an emerging technology with the environmental and economic advantages in dealing with reactive intermediates or energetic materials to achieve the continuous “scaled out” processes in chemical and pharmaceutical industries [8,9,10,11,12]. At present, the efforts on the utilization of the continuous flow process in Knoevenagel condensation reaction have been reported. For example, Wiles et al. [13] utilized 3-(1-piperazino)propyl-functionalized silica gel as the catalyst, and EOF-based micro-fabricated device as the flow reactor for Knoevenagel condensation reaction, presenting high yields (>99%) and product purity in all cases. Zhang et al. [14] have fabricated a stable ZIF-8/NaA composite membrane microreactor for the continuous flow Knoevenagel condensation, achieving nearly 100% product yield in a short residence time under mild conditions. Tsutsumi et al. [15] prepared a basic nanofibrillar chitin aerogel as the renewable catalyst for Knoevenagel condensation, which presented high performance in flow mode. Xu et al. [16] designed a basic organocatalyst through the immobilization of DABCO on polystyrene support. The prepared polystyrene-immobilized DABCO catalyst presents the high catalytic activity in both batch and continuous flow reactor with excellent selectivity and short reaction time. Therefore, the utilization of the solid amine catalyst for the continuous flow Knoevenagel condensation reaction is a promising way to the synthesis of the high value-added chemicals. The solid amine catalysts are commonly prepared through the functionalization of the support with –NH_2_ group. The supports are classified into molecular sieves [17,18], metal organic framework [19,20,21,22], polymers [16,23], graphene oxide [24,25], and others [26,27,28,29]. However, the utilization of most of these supports suffers from one or more drawbacks such as the harsh preparation conditions, equipment corrosion, environmental pollution, expensive reagents used, tedious workup procedures, excessive dependency on fossil resource, etc. [16,17,18,19,20,21,22,23,24,25,26,27,28,29]. Herein, the support originated from the renewable feedstock will be one of the promising candidates for the preparation of solid amine catalysts in the field of the continuous production of high value-added chemicals through Knoevenagel condensation reaction.

Sugarcane bagasse (SCB), as a typical agriculture waste, contains a lot of hydroxyl groups (–OH) due to its primary components of cellulose (40−50%), hemicellulose (25−35%), and lignin (15−20%) [30]. It is an ideal renewable catalyst support for the fabrication of the solid catalyst through a simple amine silanization process. Herein, based on our previous studies on the utilization of the biomass resource [31,32,33], an amine-functionalized sugarcane bagasse, denoted as SCB-NH_2_, has been designed and utilized in the continuous flow Knoevenagel condensation reaction at room temperature. The Knoevenagel condensation between benzaldehyde and malononitrile was set as the model reaction in batch and flow reaction systems. The physicochemical properties and catalytic performance of SCB-NH_2_ in batch and continuous flow conditions have been investigated carefully.

## 2. Results and Discussion

### 2.1. Characterization of SCB-NH_2_

**Figure 1:**
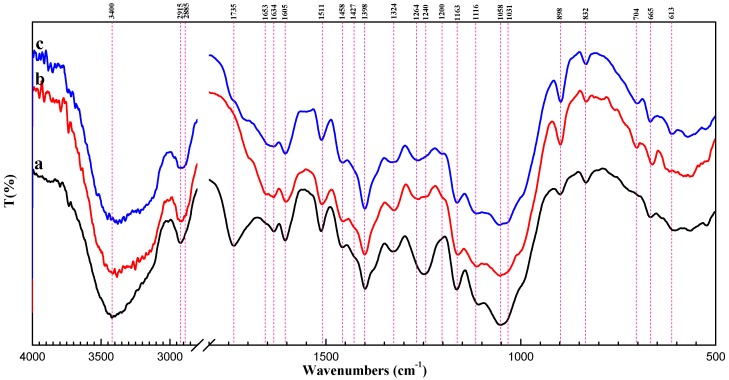
FT-IR spectra of SCB (**a**), fresh SCB-NH_2_ (**b**), and recovered SCB-NH_2_ (**c**).

Fourier transform infrared (FT-IR) spectra of primary SCB and SCB-NH_2_ are shown in Figure 1. Peaks at 3400, 2915 and 2885 cm^−1^ for SCB are due to stretching of –OH, –CH_3_, and –CH_2_ groups, respectively (Figure1a) [34]. Peaks at 1427, 1398, 1163, 1058, and 898 cm^−1^ are assigned to the characteristic vibrations of cellulose [35]; and the peak at 1735 cm^−1^ is assigned to the stretching vibration of acetyl groups attached to hemicellulose in the raw material [36]. Signals at 1634, 1605, 1511, 1458, 1324, 1264, 1200, 1116, and 832 cm^−1^ are ascribed to the characteristic vibrations of aromatic rings in lignin [37]. The results are in agreement with the primary component of SCB (Table S1).

After the amination of SCB with (3-chloropropyl)trimethoxysilane (CTEOS) and ethylenediamine (Figure 1b), the absorbance at 1735 cm^−1^ for acetyl group in the spectrum of SCB-NH_2_ disappeared, implying the completed deacetylation of hemicellulosic fraction under the alkaline condition for the amination process [38]. Furthermore, from the spectra of SCB before and after the amination, no bands assigned to N–H stretching and bending are observed in the range of 3500–3000 cm^−1^ and 1700–1300 cm^−1^ beside the weak absorbance at 1653 cm^−1^. This could be caused by the strong absorbance of hydroxyl group which overlaps the absorbance of N-H from SCB-NH_2_. The absorbance at 1653 cm^−1^ was assigned to the deformation vibrations of secondary amines [39], confirming the successful animation of SCB with amine group (–NH_2_).

To deeply understand the amine content, the SCB and SCB-NH_2_ were also analyzed by elemental analysis (Table 1). Before the amination, the nitrogen element (N) content in SCB is only 0.19%, which is attributed to a minor amount of protein in the primary SCB [40,41,42]. After the amination, the N content increases to 1.98%, confirming the successful grafting of –NH_2_ groups onto SCB. Comparing with the N content in SCB, the increment of 1.79% in the N content in SCB-NH_2_ corresponds to 1.28mmol of –NH_2_ group in 1 g of SCB-NH_2_ catalyst. Combined with the result of FT-IR and elemental analysis, the successful preparation of SCB-NH_2_ is further verified.

**Figure 2:**
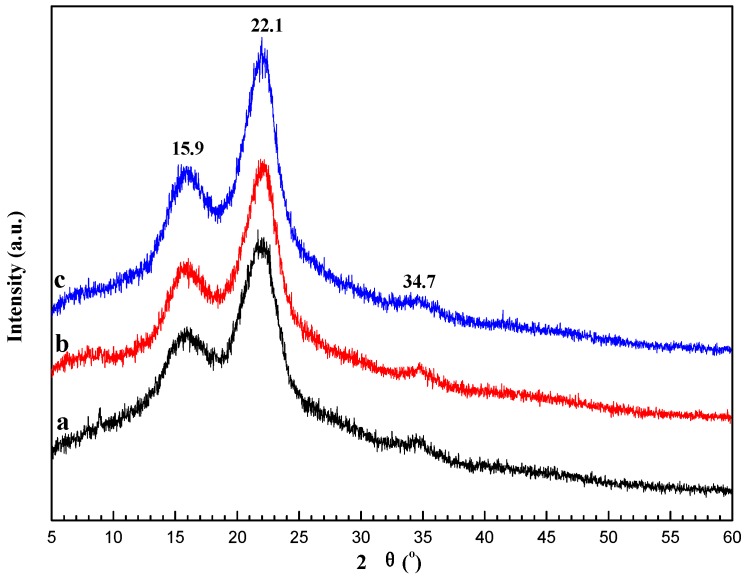
XRD patterns of SCB (**a**), fresh SCB-NH_2_ (**b**), and recovered SCB-NH_2_ (**c**).

To evaluate the stability of SCB in the amination process, the crystalline structure of SCB before and after the amination was investigated through powder X-ray diffraction (XRD) methods (Figure 2). For SCB, three peaks at 15.9, 22.1, 34.7° are observed, which are attributed to cellulose I crystal [35]. These peaks are also observed in the XRD pattern of SCB-NH_2_, indicating SCB is stable in this two-step amination process.

**Figure 3:**
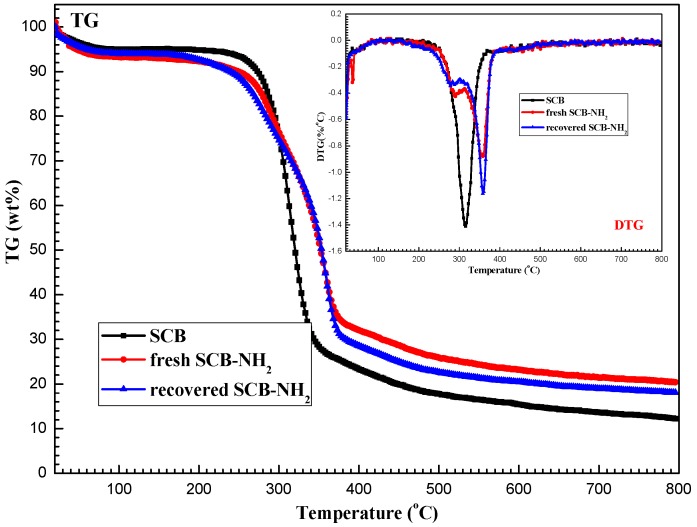
Thermal decomposition profiles of SCB, fresh SCB-NH_2_, and recovered SCB-NH_2_.

The thermal stabilities of SCB and SCB-NH_2_ were also investigated by thermogravimetry analysis method (TG). The thermal decomposition profiles are presented in Figure 3. In all samples, the first weight loss stage below 100 °C was ~5%, which is associated with the evaporation of water adsorbed in the samples. As for SCB sample, the second weight loss stage (~64%) started at 250 °C and then proceeded rapidly below 350 °C. At 800 °C, refractory constituents (~12%) such as tar and/or char and inorganic salts remained. Weight loss peaking at 315 °C in the differential thermogravimetry (DTG) curve indicates the rapid decomposition of hemicellulose, cellulose and lignin in raw material [43]. The second weight loss stage of SCB-NH_2_ began at 260 °C and proceeded rapidly to the third weight loss stage at 325 °C with weight loss of 20% and 35% respectively, leaving refractory constituents (~20%) containing tar and/or char, inorganic salts, and SiO_2_ at 800 °C [44]. The characteristic weight-loss peaks at 287 °C and 360 °C is due to the rapid decomposition of hemicellulose and grafted groups (–NH_2_, –(CH_2_)_n_–, etc.). The weight-loss at 360 °C is possible caused by the combination of cellulose and lignin. This process for SCB-NH_2_ is 45 °C higher than those in raw material showed above. This implies the protection effect of silicane coupling agent on SCB, which further illustrates that SCB support is stable in amination process.

**Figure 4:**
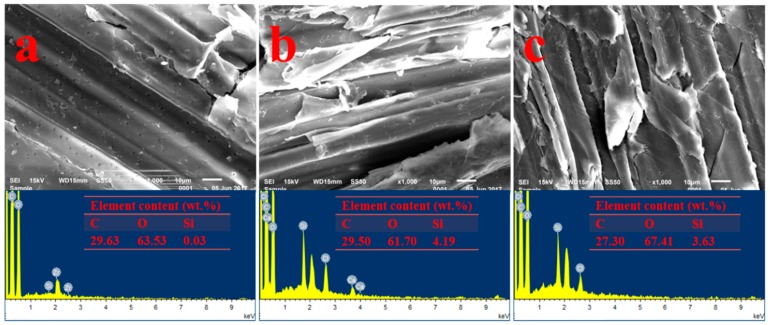
SEM images of SCB (**a**), fresh SCB-NH_2_ (**b**), and recovered SCB-NH_2_ (**c**).

The morphologies and surface characteristics of SCB and SCB-NH_2_ were also investigated by scanning electron microscope (SEM) and energy dispersive X-ray (EDX). The SEM images clearly showed that SCB possessed a smooth surface and compact morphology (Figure 4a). After being grafted with CTEOS and ethylenediamine, the surface was still smooth (Figure 4b), indicating the uniform distribution of silicane coupling agent on SCB. The direct evidence for the silicane coupling agent in SCB-NH_2_ is that silicon content increases to 4.16%. Together with FT-IR and elemental analysis, the results from SEM and EDX further confirm the successful preparation of SCB-NH_2_.

Herein, on the basis of the above results, the as-prepared solid amine catalyst (SCB-NH_2_) with the –NH_2_ group as the active site is a constitutionally stable catalyst in the base catalysis field. The utilization of SCB-NH_2_ as the base catalyst for Knoevenagel condensation in batch and continuous flow processes was investigated in the following section.

### 2.2. Catalytic Performance in Batch Reaction

Before the investigation of the continuous flow Knoevenagel condensation, the catalytic performance of SCB-NH_2_ was firstly investigated in batch conditions. Table 2 showed the effect of catalyst dosage at room temperature (25 °C) on the reaction for the full conversion of benzaldehyde monitored by thin layer chromatography (TLC). In the absence of any catalyst, the reaction time was 10 h (Table 2, entry 1). After adding SCB-NH_2_, the reaction time reduced rapidly. When the amount of the added catalyst increased from 0.05 to 0.30 g, the reaction time also decreased from 58 to 9 min (Table 2, entries 2–7). Noticeably, the yields of the isolated product in different reaction conditions were still higher than 80%, implying the coexistence of side-reactions neglected. The catalytic performance of SCB and SCB-Cl for this base-catalyzed Knoevenagel condensation was also investigated (Table 2, entries 8 and 9). The results showed that both of two catalysts had certain catalytic activities for this condensation due to the activation effect of hydroxyl groups (–OH) attached to the surface of SCB and SCB-Cl through the intermolecular hydrogen-bond effect [26,45]. However, the reaction time by using either SCB or SCB-Cl was still much longer than that for SCB-NH_2_ catalyst, demonstrating the –NH_2_ group was the dominant active center in SCB-NH_2_ catalyst.

The solvent effect on this reaction was also investigated. When no solvent was added into the catalyst-free system (Table 2, entry 10), the condensation reaction proceeded very slowly, the reaction time for the full conversion is as long as 28 h. To understand the influence of solvent on this condensation reaction in depth, the solvent types and composition have been studied extensively (Table 3). From the green and economic chemistry point of view, the low boiling point and toxicity solvents, such as tetrahydrofuran (THF), acetone, methanol (MeOH), ethanol (EtOH) as outlined in the Pfizer solvent selection guide [46,47], have been selected as the “preferable” solvents.

Table 3 showed that the molecular structure and composition of solvent had the important impact on the catalytic performance of SCB-NH_2_ in this condensation reaction. The condensation reaction was completed in only 10 min under the solvent-free system (Table 3, entry 1). However, when the aprotic solvents such as THF or acetone were added into the reaction system, the reaction proceeded very slowly. After 24 h there was still ~50% of benzaldehyde remained in the reaction system (Table 3, entries 2 and 3), demonstrating the unfavorable effect of aprotic solvents on this reaction. Subsequently, the protic MeOH and EtOH were used as the solvents. The catalytic performance of SCB-NH_2_ improved in a certain degree. However, the reaction time still reached up to about 60 min with more than 90% product yield (Table 3, entries 4 and 5). The water contained in the solvent or formed during this reaction facilitated the condensation process [48,49]. By using co-existence of water with protic solvent (EtOH), the catalytic performance of SCB-NH_2_ elevated significantly accompanying with ~95% product yield. When 98% of EtOH was used as the solvent, the reaction time reduced to 25 min (Table 3, entry 6), which was much shorter than that in anhydrous solvent (57 min). Further increase of water content to 20% in the solvent, the reaction time for the full conversion of benzaldehyde is much shorter and reduced to 9 min (Table 3, entries 7–9). 

However, as the water content increased, the solubility of product in the solvent decreased accordingly (Table S2). When the water content was more than 5%, a certain amount of white solid was crystallized out before the reaction is completed (Figure S1), which limited the subsequent continuous flow reactions. Hence, on the basis of the results above, the commercially available 95% EtOH was selected as the preferred solvent for the continuous flow reaction. It should be mentioned that the solubility of product in 95% EtOH was 0.38 g/10 mL, which was in accordance with the theoretical product concentration in 20 mL 95% EtOH with 5 mmol of benzaldehyde and malononitrile as the starting materials.

In summary, considering the reaction time and product yield, the amount of catalyst added was 0.15−0.3 g and the reaction time ranges from 9 to 15 min. The optimized batch was used as a guide for setting the residence time in continuous flow conditions with 95% EtOH as the solvent.

### 2.3. Continuous Flow Knoevenagel Condensation Reaction

Based on the results from batch conditions, the concentration of raw material in the subsequent continuous flow reactions was 0.25 mmol/mL and the reaction was carried out in 95% EtOH solvent at room temperature. The reactor is shown in Figures S2 and S3, and the amount of the catalyst filled in reactor was about 0.9 g. The retention volume was about 5 mL determined by the volume method. According to the suggested residence time of 9~15 min in the batch reaction, the flow rate was firstly set as 0.5 mL/min.

As the flow rate was controlled at 0.5 mL/min, the complete conversion of benzaldehyde was achieved (Figure S4). This means the better catalytic performance of SCB-NH_2_ in continuous flow condition than that in batch condition at the same reaction time. The production capacity is an important criterion for evaluating the usability of continuous process. Hence, the higher flow rate in this flow process had been investigated inevitably. As the flow rate increased to 1.5 mL/min, the conversion of benzaldehyde was still as high as 99.5% and the product yield is over 99% (Figure S4). However, as the flow rate was more than 1.5 mL/min, the catalytic performance of this continuous system declined significantly. At the flow rate of 3.0 mL/min, the conversion of benzaldehyde was only 70.4%, indicating its weak capability on catalytic conversion of a large amount of benzaldehyde in the relative short reaction time (residence time). It was noted that, the selectivity of product in all flow conditions was still more than 99.5%, demonstrating the perfect conversion of benzaldehyde to product. Based on the above results, 1.5 mL/min is the preferred flow rate for this continuous flow process, and was used to evaluate the tolerability of SCB-NH_2_ in time course (catalyst lifetimes).

The outflow solution was analyzed through nuclear magnetic resonance (NMR) and gas chromatography (GC) (Figures S5 and S6), and the results were given in Figure S7. It is demonstrated that no obvious deactivation was observed. The product yield and selectivity were over 97% and 99% after 80 h, respectively, indicating its excellent catalytic stability. After the volatilization of solvent, the crude product was obtained as the white solid (Figure S8). Furthermore, the crude product was easily purified through recrystallization process in 95% EtOH, and the purity in the final product is over 99% (Figure S9).

**Scheme 1:**
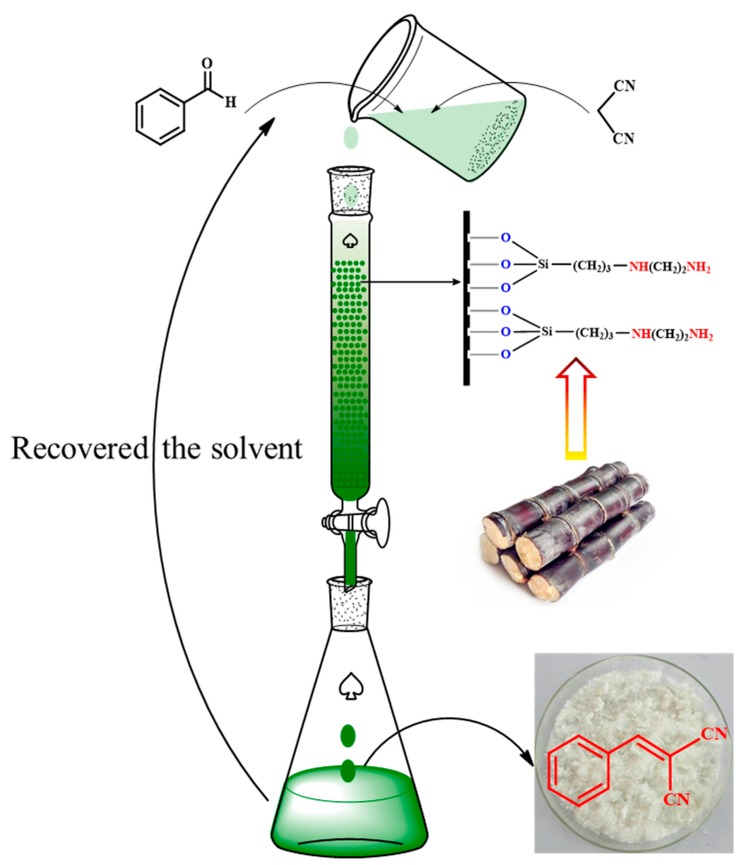
Schematic diagram of continuous flow Knoevenagel reaction at room temperature.

The catalyst was recovered after 120 h of flow reaction time, and further characterized through FT-IR (Figure 1c), elemental analysis (Table 1), XRD (Figure 2c), TG-DTG (Figure 3c), and SEM (Figure 4c). These results showed that the support (SCB) was very stable in this flow conditions. The slight deactivation after 80 h is attributed to the gradual loss of amine groups under flow conditions, which is proved by the elemental analysis. Therefore, the results above demonstrated that the as-prepared SCB-NH_2_ was a stable and efficient renewable catalyst for Knoevenagel condensation reaction under continuous flow conditions at room temperature (Scheme 1).

Furthermore, the applicability of the SCB-NH_2_ catalyzed continuous flow Knoevenagel condensation reaction was also tested with different aldehydes. It was found that the glass chromatography column filled with SCB-NH_2_ was not a suitable reactor for Knoevenagel reaction of furfural with malononitrile due to the rapid reaction rate and limited flow rate restricted by gravitational flow (Figure S10a). To realize the controllable condition, a waste chromatographic column installed in a high-performance liquid chromatography (HPLC) was used as a reactor instead of the glass reactor (Figure S10b), where the reaction condition, especially flow rate, can be controlled by HPLC instrument exactly (Figure S10b). Subsenquently, the resulting brown product from Knoevenagel condensation of furfural and malononitrile can be obtained after the recovery of solvent (Figure S10d). The results showed that the SCB-NH_2_ could be a potential substrate-tolerable catalytic material if a continuous reactor with controllable reaction conditions was adopted.

## 3. Materials and Methods 

Benzaldehyde, malononitile, (3-chloropropyl)trimethoxysilane, and ethylenediamine were obtained from the J & K Chemical Company; THF, acetone, MeOH, EtOH, and other agents were all purchased from Guangzhou Chemical Reagent Factory. The purity of these chemicals is more than 98% and used without purification.

SCB was obtained from local Farm (Guigang, Guangxi Province, China). The primary ingredients of these SCB were analyzed according to TAPPI test method and listed in Table S1. Prior to use, SCB were dried under vacuum at 80 °C for 24 h. After being milled and sieved, the powder with particle sizes between 40–60 mesh was selected. 

### 3.1. Preparation and Characterization of SCB-NH_2_

**Scheme 2:**
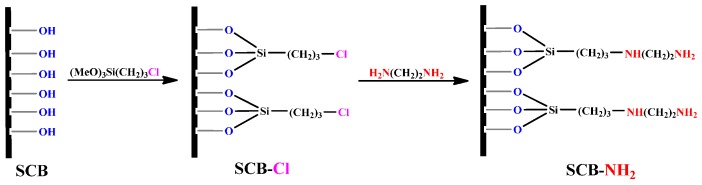
Preparation of SCB-NH_2_.

As shown in Scheme 2, SCB-NH_2_ is first prepared by treating the dried SCB with (3-chloropropyl)trimethoxysilane (CTEOS) (step I) and then modified with ethylenediamine through the amination (step II) [50,51,52,53]. In a typical procedure, dried SCB (2.0 g) was first added into anhydrous toluene (100 mL), then 1.0 g (~5.0 mmol) of (3-chloropropyl)trimethoxysilane and 0.1 mL (~5.5 mmol) of H_2_O were added to this suspension, the mixture was further stirred and heated to reflux for 4 h, subsequently, 1.5 g (~25mmol) ethylenediamine was added dropwise. After being refluxed for another 4 h, the mixture was filtrated and washed by EtOH for three times and dried in vacuum oven at 80 °C for 24 h. The obtained material was denoted as SCB-NH_2_.

The as-prepared catalyst was characterized by FT-IR, element analysis, XRD, TG and SEM-EDX. FT-IR spectroscopy was performed on a Thermo Nicolet 6700 spectrometer (Thermo Electron Corp., Waltham, MA, USA). The samples and KBr (spectral purity) were mixed at a ratio of 2:98 (*w*/*w*), ground, and pressed into a disc; then the FT-IR spectra were recorded using the disk in the range of 4000–400 cm^−1^ with a resolution of 4 cm^−1^. Elemental composition was analyzed through Vario EL cube Elemental analyzer (Elementar, Germany) to determine the content of the C, H, N elements presented in each sample and the amount of amine functionalities grafted onto SCB; the elemental analysis was repeated three times to minimize error. XRD patterns (2θ = 5–60°) were measured on a Bruker AXS D8 Advance instrument (Karlsruhe, Germany) with Nickel-filtered CuKα (wavelength = 0.154 nm) at 40 kV and 40 mA. The samples were mounted on a holder, and the proportional counter detector was set to collect data at a rate of 2θ = 4°/min with increments of 0.04° for 2θ values. TG profiles were measured on a Sta449 F3 thermogravimetric analyzer (Netzsch, Germany) using N_2_ flow of 50 mL/min and a protective N_2_ flow of 30 mL/min. The samples are weighed about 10–15 mg, and were heated from 50 to 800 °C at a heating rate of 10 °C/min. SEM images were recorded with a JSM-6510LV SEM instrument (JEOL, Tokyo, Japan) with the combined EDX analyzer operated at an accelerating voltage of 15 kV in a high-vacuum mode; prior to SEM observation, the samples were coated with gold by sputtered deposition using a Quick coater SC-701 (Sanyu Electron, Tokyo, Japan).

### 3.2. Typical Batch Reaction

Before the continuous flow reaction, the reaction condition was optimized in batch system. A 50 mL round bottomed flask was charged with 5.0 mmol benzaldehyde, 5.0 mmol malononitrile, 20 mL 95% EtOH, and 0.15 g SCB-NH_2_ catalyst. The reaction mixture was stirred at room temperature (25 °C). The formation of the products was monitored by TLC, which was performed by using commercially available 100–400 mesh silica gel plates (GF254) with the visualization at 254 nm (3:1 (*v*/*v*) petroleum ether (60–90 °C)/ethyl acetate as the eluting agent). After reaction, the catalyst was filtered off and washed with 95% EtOH (3 × 10 mL). The white crystals as the target product was obtained after recovery of the EtOH from the resulting filtrate. The product yield was calculated through comparing the weight of obtained product with theoretical value. To minimize errors, all experiments for batch conditions were repeated three times to calculate the mean values and standard deviations.

The structure and purity of product were analyzed by ^1^H nuclear magnetic resonance (^1^HNMR, Bruker DRX 400, CDCl_3_ as the solvent with 0.03% TMS as internal standard) and gas chromatography (GC, Shimadzu QP 2010, capillary column (30 m × 0.25 mm, 0.25 μm), FID detector and nitrogen as the carrier (flow rate of 1 mL/min); the injection volume was 0.1 μL; programmed oven temperature: hold at 100 °C for 1.0 min, then ramped up to 220 °C with the heating rate of 20 °C/min, then, hold at this temperature for 1.0 min; injector: kept at 250 °C in spit mode with spit ratio of 30:1).

### 3.3. Continuous Flow Reaction

Continuous flow Knoevenagel condensation was carried out at room temperature (25 °C) in a continuous flow glass chromatography column as shown in Figure S2. About 0.9 g of SCB-NH_2_ catalyst was charged into the glass column (Figure S3), then the reactant mixture with equimolar benzaldehyde and malononitrile pre-dissolved in 95% EtOH was added by a dropping funnel, and the flow rate was controlled by the stopcock of glass column. The product was collected in a flask and analyzed by ^1^H NMR and GC at each given time interval. After the recovery of the EtOH, a large amount of the white solid product was obtained. All experiments were replicated at least three times; the standard measurement uncertainties for NMR and GC analysis were less than ±0.2% according to the repeated detection results.

## 4. Conclusions

Herein, a renewable catalyst, viz., SCB-NH_2_, was prepared successfully through a two-step amination process. The as-prepared SCB-NH_2_ exhibited high catalytic performance in liquid phase continuous flow Knoevenagel condensation at room temperature. At the flow rate of 1.5 mL/min, the as-constructed continuous flow system presented longer lifetimes (~80 h) with excellent activity for perfect conversion of benzaldehyde into the product. It is believed that this renewable SCB-NH_2_ with excellent performance possesses potential and broad prospects in organic synthesis or catalysis. Hence, the results showed the SCB-NH_2_ is a promising catalyst for the synthesis of fine chemicals by Knoevenagel condensation reaction in large scale, and also confirmed the modification of renewable SCB with –NH_2_ group is a potential avenue for the preparation of amine-functionalized catalytic materials in industry. Moreover, by using the solid amine catalyst described here, further studies are currently underway in our group to extend both the substrates and reaction types such as Henry reaction, Michael addition, multicomponent reactions, etc., enabling more complex syntheses to be demonstrated in the future.

## Figures and Tables

**Table 1:** The element analysis of SCB, fresh SCB-NH_2_, and recovered SCB-NH_2_.

Samples	Element Content (wt. %)		
	C	H	N
SCB	41.52 ± 0.22	7.97 ± 0.07	0.19 ± 0.02
Fresh SCB-NH_2_	41.24 ± 0.30	8.00 ± 0.05	1.98 ± 0.01
Recovered SCB-NH_2_	41.74 ± 0.24	7.98 ± 0.06	1.82 ± 0.01

**Table 2:** The influence of the catalyst dosage ^a^.

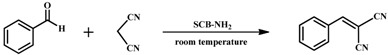			
**Entry**	**Catalyst Dogase (g)**	**Time (min)**	**Yield (%) ^b^**
1	—	10 ± 0.5 (h)	89.2 ± 2.4
2	0.05	58 ± 5	92.9 ± 3.1
3	0.10	33 ± 3	94.3 ± 2.8
4	0.15	15 ± 2	95.1 ± 2.6
5	0.20	12 ± 2	94.1 ± 3.2
6	0.25	10 ± 1	93.8 ± 2.9
7	0.30	9 ± 1	94.3 ± 2.1
8 ^c^	0.15	4 ± 0.2 (h)	94.9 ± 2.6
9 ^d^	0.15	4 ± 0.2 (h)	87.9 ± 2.7
10 ^e^	—	28 ± 1.5 (h)	85.2 ± 2.8

^a^ Reaction conditions: 5.0 mmol of benzaldehyde, 5.0 mmol of malononitrile, 20 mL of 95% EtOH, room temperature (25 °C); ^b^ Isolated yield; ^c^ SCB as the catalyst; ^d^ SCB-Cl as the catalyst; ^e^ Without solvent.

**Table 3:** The influence of the solvent ^a^.

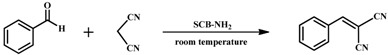			
**Entry**	**Solvent**	**Time (min)**	**Yield (%) ^b^**
1	—	10 ± 1	95.2 ± 1.9
2 ^c^	THF	>24 (h)	51.5 ± 2.1
3 ^c^	Acetone	>24 (h)	54.3 ± 3.2
4	MeOH	66 ± 3	93.3 ± 2.7
5	EtOH	57 ± 3	94.1 ± 2.5
6	98% EtOH	25 ± 2	94.3 ± 2.8
7	95% EtOH	15 ± 2	95.1 ± 2.6
8	90% EtOH	10 ± 1	94.1 ± 2.3
9	80% EtOH	9 ± 1	93.8 ± 3.1

^a^ Reaction conditions: 5.0 mmol of benzaldehyde, 5.0 mmol of malononitrile, 0.15 g of SCB-NH_2_, 20 mL of solvent, room temperature (25 °C); ^b^ Isolated yield; ^c^ the yield was determined by GC due to the incomplete conversion of benzaldehyde.

## Supplementary Materials

The following are available online, Table S1: Primary ingredients of sugarcane bagasse, Table S2: The solubility of products in different solvent system, Figure S1: The photography of reaction mixture, Figure S2: The glass chromatography column, Figure S3: The photography of continuous flow reactor, Figure S4: The influence of flow rate on conversion and yield, Figure S5: ^1^H NMR spectra of the products solution at different flow time, Figure S6: The GC profiles of outflow, Figure S7: Continuous flow Knoevenagel reaction at room temperature, Figure S8: The crude product obtained after recovered solvent, Figure S9: ^1^H NMR spectra of the purified product, Figure S10: The continuous flow Knoevenagel condensation of furfural with malononitrile.
