# Supplementary material for: Amine-Functionalized Sugarcane Bagasse: A Renewable Catalyst for Efficient Continuous Flow Knoevenagel Condensation Reaction at Room Temperature
Source: Molecules. 2017 Dec 24;23(1):43. doi: 10.3390/molecules23010043 (PMC5943943; doi:10.3390/molecules23010043)
Supplement: Supplementary file 1 [file molecules-23-00043-s001.docx]

Supplementary material

Amine-functionalized Sugarcane Bagasse: A Renewable Catalyst for Efficient Continuous Flow Knoevenagel Condensation Reaction at Room Temperature

Yanhui Qiao ^1,2^, Junjiang Teng ^2^, Shuangfei Wang ^1,3^* and Hao Ma ^2,^*

^1^ School of Chemistry and Chemical Engineering, Guangxi University, Nanning 530004, China; [qyhmmc@gdupt.edu.cn](mailto:qyhmmc@gdupt.edu.cn) (Y.Q.); [wangsf@gxu.edu.cn](mailto:wangsf@gxu.edu.cn) (S.W.)

^2^ College of Chemical Engineering, Guangdong University of Petrochemical Technology, Maoming 525000,China; [tjjteng@gdupt.edu.cn](mailto:tjjteng@gdupt.edu.cn) (J.T.); [thma@gdupt.edu.cn](mailto:thma@gdupt.edu.cn) (H.M.)

^3^ College of Light Industry and Food Engineering, Guangxi University, Nanning 530004, China.

***** Correspondence: [wangsf@gxu.edu.cn](mailto:wangsf@gxu.edu.cn) (S.W.); [thma@gdupt.edu.cn](mailto:thma@gdupt.edu.cn) (H.M.)

Contents of the supplementary material:

Primary ingredients of sugarcane bagasse…………………………………………………………...Table S1

The solubility of products in different solvent system…...…………………………………………Table S2

The photography of reaction mixture………………………………………………………………Figure S1

The glass chromatography column…………………………………………………………………Figure S2

The photography of continuous flow reactor…………....…....…....………………………………Figure S3

The influence of flow rate on conversion and yield ………………………………………………Figure S4

^1^H NMR spectra of the products solution at different flow time…………………………………Figure S5

The GC profiles of outflow…………....…....…....……………………………………………………Figure S6

Continuous ﬂow Knoevenagel reaction at room temperature……………………………………Figure S7

The crude product obtained after recovered solvent………………………………………………Figure S8

^1^H NMR spectra of the purified product…....…....…....……………………………………………Figure S9

The continuous flow Knoevenagel condensation of [furfural](javascript:void(0);) with malononitrile…………………Figure S10

**Table S1.** Primary ingredients of sugarcane bagasse

| **Ingredients** | **Cellulose** | **Hemicellulose** | **Lignin** | **Others** |
| --- | --- | --- | --- | --- |
| Content (wt%) | 44.53 ± 2.65 | 28.15 ± 1.94 | 20.21 ± 1.58 | 7.11 ± 0.14 |

**Table S2.** The solubility of products in different solvent system^a^

| **Entry** | **Solvent** | **Solubility (g/10 mL)** |
| --- | --- | --- |
| 1 | THF | 5.44 ± 0.02 |
| 2 | Acetone | 6.57 ± 0.01 |
| 3 | MeOH | 1.00 ± 0.03 |
| 4 | EtOH | 0.53 ± 0.02 |
| 5 | 98% EtOH | 0.42 ± 0.02 |
| 6 | 95% EtOH | 0.38 ± 0.03 |
| 7 | 90% EtOH | 0.24 ± 0.02 |
| 8 | 80% EtOH | 0.10 ± 0.03 |

^a^ at room temperature (25 °C)


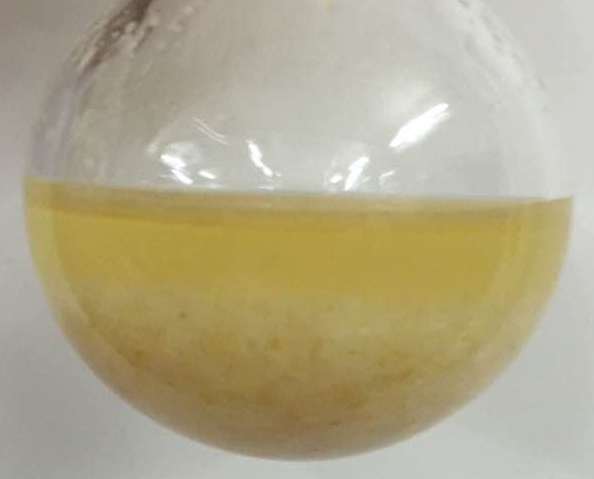


**Figure S1.** The photography of reaction mixture


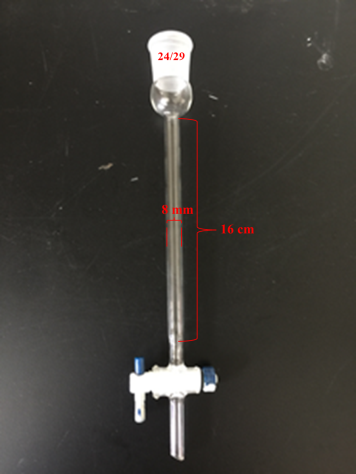


**Figure S2.** The glass chromatography column


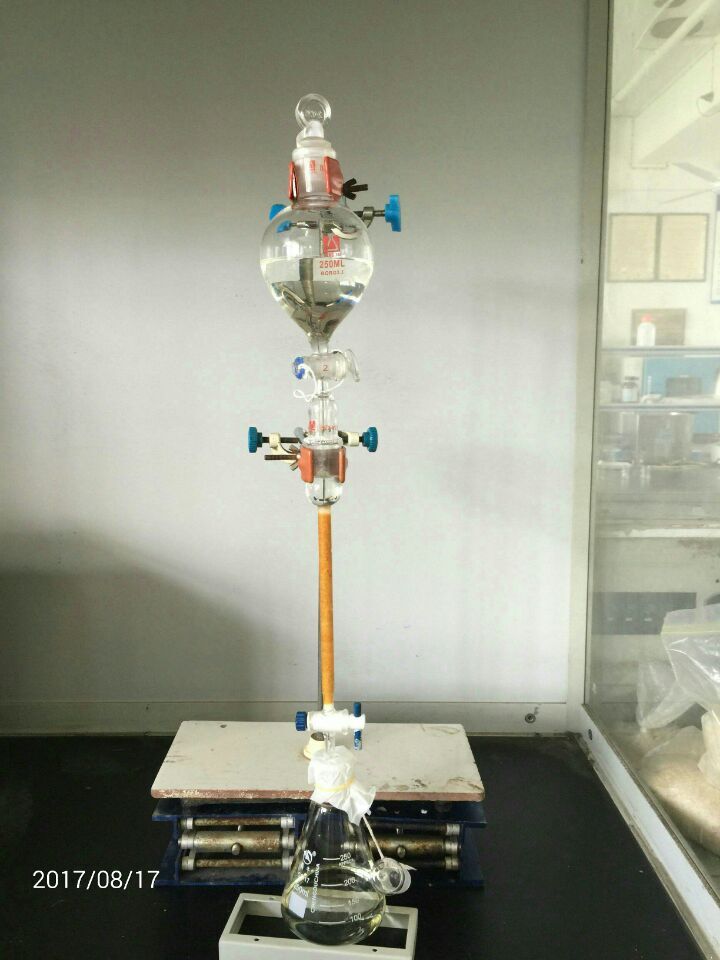


**Figure S3.** The photography of continuous flow reactor

**Figure S4.** The influence of flow rate on conversion (**a**) and yield (**b**) (Condition: the concentration of benzaldehyde and malononitrile in 95% EtOH are 0.25 mmol/mL at room temperature)

**Figure S5.** ^1^H NMR spectra of the products solution at different flow time (CDCl_3_ as the solvent)

**Figure S6.** The GC profiles of outflow (a, 40 h, b: 120 h)

**Figure S7.** Continuous ﬂow Knoevenagel reaction at room temperature (Condition: the concentration of benzaldehyde and malononitrile in 95% EtOH are 0.25 mmol/mL at room temperature (25 °C); the flow rate is 1.5 mL/min)


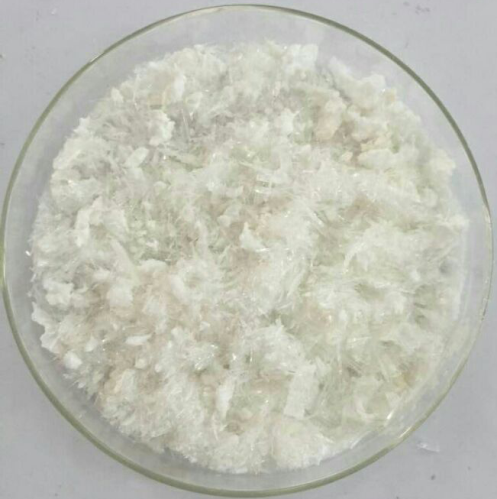


**Figure S8.** The crude product obtained after recovered solvent

**Figure S9.** ^1^H NMR spectra of the purified product (CDCl_3_ as the solvent)

**
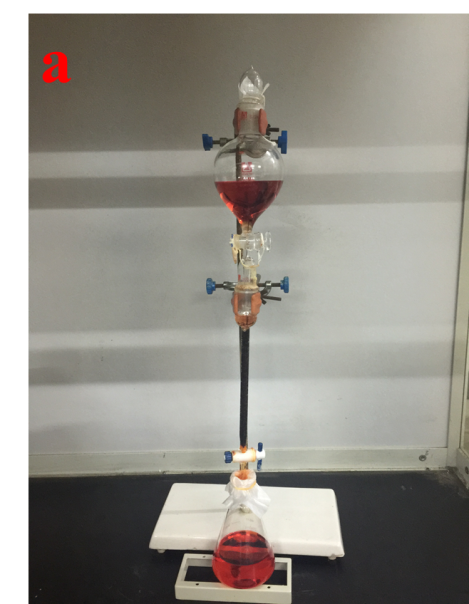

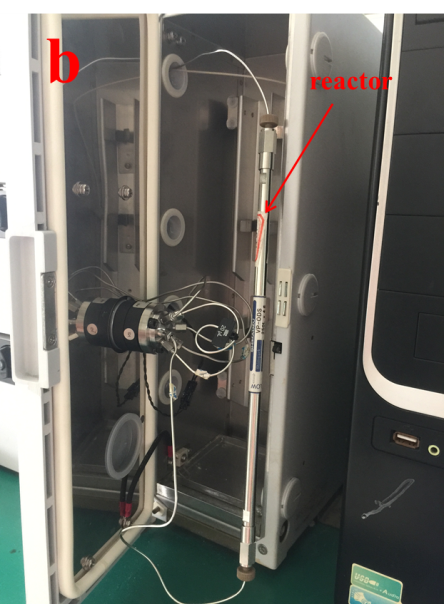

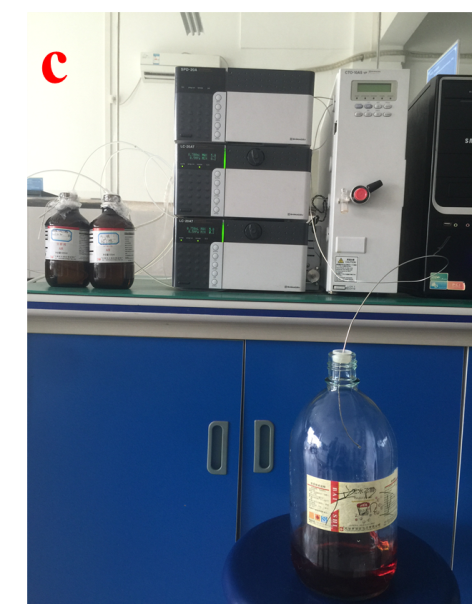
**

**
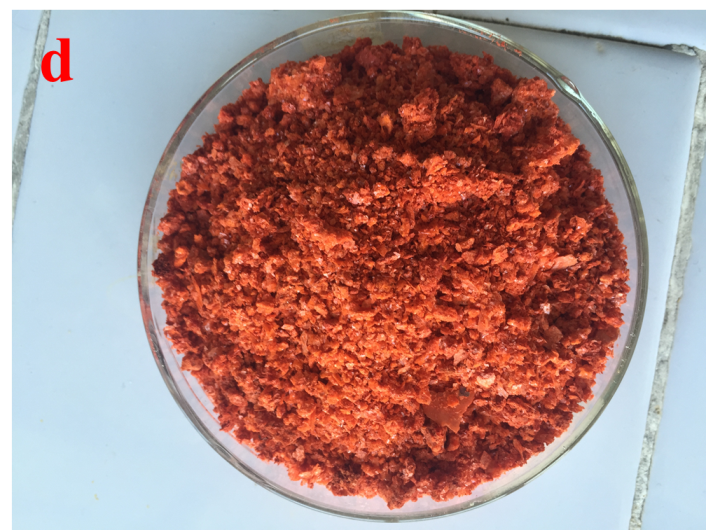
**

**Figure S10**. The continuous flow Knoevenagel condensation of [furfural](javascript:void(0);) with malononitrile

(a): the glass chromatography column reactor; (b) the HPLC [chromatographic](javascript:void(0);) [column](javascript:void(0);) reactor (Column：250 ×4.6 mm; catalyst dosage: ~0.65g; retention volume: ~2mL); (c) the continuous flow Knoevenagel condensation in HPLC [chromatographic](javascript:void(0);) [column](javascript:void(0);) reactor (Condition: the concentration of fufural and malononitrile in 80% EtOH are 0.67 mmol/mL, respectively; flow rate for both solution is 1.0 mL/min at room temperature；LC-20AT，SHIMADZU); (d) the obtained product after recovery of solvent
